# Targeting MYC at the intersection between cancer metabolism and oncoimmunology

**DOI:** 10.3389/fimmu.2024.1324045

**Published:** 2024-02-08

**Authors:** Simran Venkatraman, Brinda Balasubramanian, Chanitra Thuwajit, Jaroslaw Meller, Rutaiwan Tohtong, Somchai Chutipongtanate

**Affiliations:** ^1^ Department of Biochemistry, Faculty of Science, Mahidol University, Bangkok, Thailand; ^2^ Division of Cancer and Stem Cells, Biodiscovery Institute, School of Medicine, University of Nottingham, Nottingham, United Kingdom; ^3^ Department of Immunology, Faculty of Medicine Siriraj Hospital, Mahidol University, Bangkok, Thailand; ^4^ Department of Environmental and Public Health Sciences, University of Cincinnati College of Medicine, Cincinnati, OH, United States; ^5^ Department of Biomedical Informatics, University of Cincinnati College of Medicine, Cincinnati, OH, United States; ^6^ Division of Biomedical Informatics, Cincinnati Children’s Hospital Medical Center, Cincinnati, OH, United States; ^7^ Milk, microbiome, Immunity and Lactation research for Child Health (MILCH) and Novel Therapeutics Lab, Division of Epidemiology, Department of Environmental and Public Health Sciences, University of Cincinnati College of Medicine, Cincinnati, OH, United States

**Keywords:** MYC, metabolism, oncoimmunology, cancer, immune evasion

## Abstract

MYC activation is a known hallmark of cancer as it governs the gene targets involved in various facets of cancer progression. Of interest, MYC governs oncometabolism through the interactions with its partners and cofactors, as well as cancer immunity via its gene targets. Recent investigations have taken interest in characterizing these interactions through multi-Omic approaches, to better understand the vastness of the MYC network. Of the several gene targets of MYC involved in either oncometabolism or oncoimmunology, few of them overlap in function. Prominent interactions have been observed with MYC and HIF-1α, in promoting glucose and glutamine metabolism and activation of antigen presentation on regulatory T cells, and its subsequent metabolic reprogramming. This review explores existing knowledge of the role of MYC in oncometabolism and oncoimmunology. It also unravels how MYC governs transcription and influences cellular metabolism to facilitate the induction of pro- or anti-tumoral immunity. Moreover, considering the significant roles MYC holds in cancer development, the present study discusses effective direct or indirect therapeutic strategies to combat MYC-driven cancer progression.

## 1 Introduction

MYC is a proto-oncogenic transcription factor that governs a myriad of cellular processes including cell proliferation, survival, DNA damage repairs, histone modifications, and cellular metabolism (1). MYC is a family of transcription factors, i.e., MYC(c-MYC), MYCN (N-Myc) and MYCL (L-Myc), all of these contain a basic helix-loop-helix structure (bHLH) and leucine zipper (LZ) structural motifs with 6 conserved regions known as the MYC homology boxes (2). MYC family shares similar functions but has distinct tissue specificity; c-MYC is ubiquitously expressed in a broad variety of tissue development, n-MYC in neural and hematopoietic tissues, and L-MYC in lungs. The bHLH structure allows the interaction of MYC with DNA, while the LZ structure allows interaction with its partner transcription factor MAX. This MYC-MAX heterodimer interacts with numerous elements to either promote or repress transcription of gene targets (3).

Dysregulation of MYC implicates a wide array of diseases including neurodegenerative diseases (4), immune disorders (5), and cancers (6). Of the known hallmarks of cancer, MYC dysregulation has been reported to result in angiogenesis (7), cell replicative immortality (8), cell invasion and migration (8), alterations in cellular energetics (9), insensitivity to growth signals (10), and evading immune recognition and programmed cell death (6, 11). Because of its multifaceted dysregulation, MYC-driven cancers are often associated with poor prognosis (12–14). The involvement of MYC in both metabolism and immune evasion is highly concerning, especially in the context of malignant transformation. MYC promotes cell proliferation under conditions that would typically prove fatal for normal cells by manipulating glucose metabolism and eluding immunosurveillance by releasing metabolites within the tumor microenvironment (TME) (15, 16). While this facet has great implications for tumor progression, it also poses a particular threat in both tumorigenesis and potential tumor recurrence (17, 18).

Estimating up to 70% of cancers are affected by MYC aberration (19, 20), MYC therefore has been perceived as one of the most valuable targets for cancer therapy. However, direct pharmacological inhibition of MYC has remained challenging due to its lack of enzymatic activity or binding sites. Hence, this has raised interest in exploring the interactome of MYC to identify druggable targets, thereby modulating MYC-dependent transcriptome. A prototype of this approach is Omomyc, a MAX-interfering peptide. Omomyc was found to halt breast cancer progression, and regressed lung cancer in preclinical models (21). Currently, clinical trials are underway to determine the safety and efficacy of this drug in non-small cell lung cancer and colorectal cancer (ClinicalTrials.gov identifier NCT04808362). The success of this proof-of-concept inhibition of protein-protein interactions of MYC encourages the development of many such small molecules in therapeutically targeting MYC.

In this review, we enumerate the recent studies that characterize the targets and partners of MYC involved in cancer metabolism and immunology. Further, we discuss current evidence of the overlap between cellular functions governed by MYC and how one function may influence another. This guides us to further unravel how MYC orchestrates cancer growth by mediating metabolism and oncoimmunology. Lastly, in the growing interest of mitigating the ‘undruggable’ nature of MYC, we discuss currently available therapeutic strategies to combat MYC, a central target in the grand scheme of cancer.

## 2 Key MYC partners and targets

**Figure 1:**
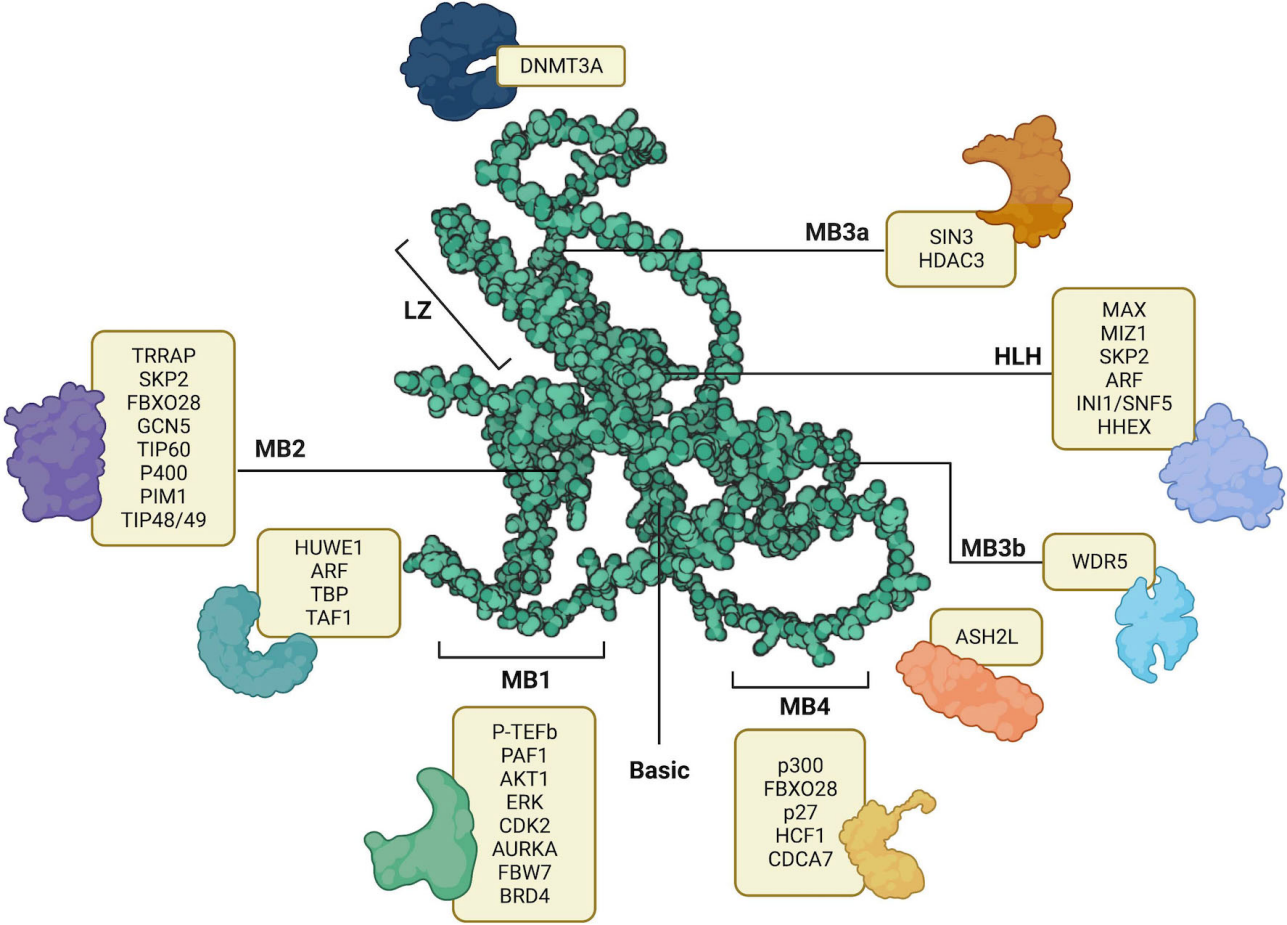
AlphaFold predicted structure of MYC (AF- P01106-F1) its annotated structural domains and their respective interactors. LZ – Leucine Zipper, HLH – Helix-Loop-Helix, MB1-MB4 – MYC binding boxes. Created with BioRender.com.

MYC structure consists of several domains that allow binding interactions of coactivators, heterodimers, or ligases. Each of these interactors facilitates the function of MYC in carrying out various biological processes. Its organization begins with a transcription activation site, which is a conserved region known as the MYC homology box (MBI and II), followed by a proline, glutamine, threonine-rich region, two more MYC homology boxes (MBIII and IV), and lastly, a basic HLH-LZ, at the carboxy-terminal (22). Because of the various regions available for interactions, and the implication of MYC in various cellular processes and molecular functions, there is a growing interest in unraveling the vast network of MYC and its interactome. In **Figure 1**, we summarized the text-mined sources of MYC protein-protein interactions with key partners.

Investigating the mechanisms of action revealed crucial insights into MYC functions; MYC utilizes its transcription activation domain to recruit cofactors containing chromatin modifiers, specifically histone acetyltransferases (HATs). One such cofactor, p300 (EP300) HAT, was identified as having a novel functional interaction with MYC (3). Moreover, p300 was also found to interact with N-MYC in regulating cell proliferation in MYCN-amplified neuroblastoma cell lines (23). Conversely, MYC transcriptionally represses gene expression of its targets by interacting with transcription factors such as MIZ-1 and NFY-B, which facilitates the recruitment of histone deacetylases (HDACs) (24). This finding highlights the multifaceted role of MYC, whereby it acts as a regulator by binding to the promoter region of target genes and modulates DNA methylation through the recruitment of HATs and HDACs.

MYC is considered a systemic regulator of diverse functions, because of the multidomain structure and the requirement of chromatin-modulating cofactors. MYC functions as a molecular switch of activating and/or repressing the transcription of its gene targets, depending on the position at which specific cofactors bind. The transactivation domain spans the MB1 and MB2 regions (22). Within this domain, cofactors that are shown to bind and activate gene transcription include FBW7 (25, 26), TAF1 (27), TBP (27), p-TEFb (28) TRRAP (3, McMahon et al., 1998), GCN5 (29), TIP60 (30), TIP48 (31), p400 (32), and SKP2 (33). These transactivating cofactors promote the transcription of target genes related to cell proliferation and survival, including CDK4 (34), CDC25A (35), and E2F1. Moreover, beyond sustained proliferative signaling, MYC has roles in various other hallmarks of cancer mediated by its gene targets. For instance, in promoting angiogenesis, MYC binds to the promoter region of VEGFA, thereby increasing its production (36). Moreover, MYC regulates invasion and migration by inducing the transcriptional activation of LGALS1 (37).

Conversely, the repression of MYC gene targets is triggered by cofactors binding in and between the regions of MB2 and MB3, and the bHLHLZ region. Cofactors that contribute to transrepression of MYC gene targets include TIP48/49, DNMT3a (38), PRC2 (39), HDAC1 (40), HDAC3 (41), KDM4B (42), and MIZ-1 (43, 44). In the initiation and progression of cancer, the expression of tumor-suppressing genes is usually repressed. Likewise, the expression of NDRG2 (45), PTEN (46), CDKN2C (46), CDKN1A (46), p21 (47, 48), p15 (48, 49), N-cadherin (48), is repressed by MYC, and therefore suppresses tumor suppressing functions, leading to cancer progression (50). Another key determinant of MYC global transcriptional amplification and systemic activity is its abundance and regulation (2). Patange et al’s investigation reveals that the overexpression of MYC results in prolonged bursts of transcriptional activation by altering the binding affinity of transcription factors involved in the pre-initiation complex to RNA polymerase II (51). Together, the abundance of MYC and a balance of these transactivators or transrepressors, dictate the fate of cancer progression.

**Figure 2:**
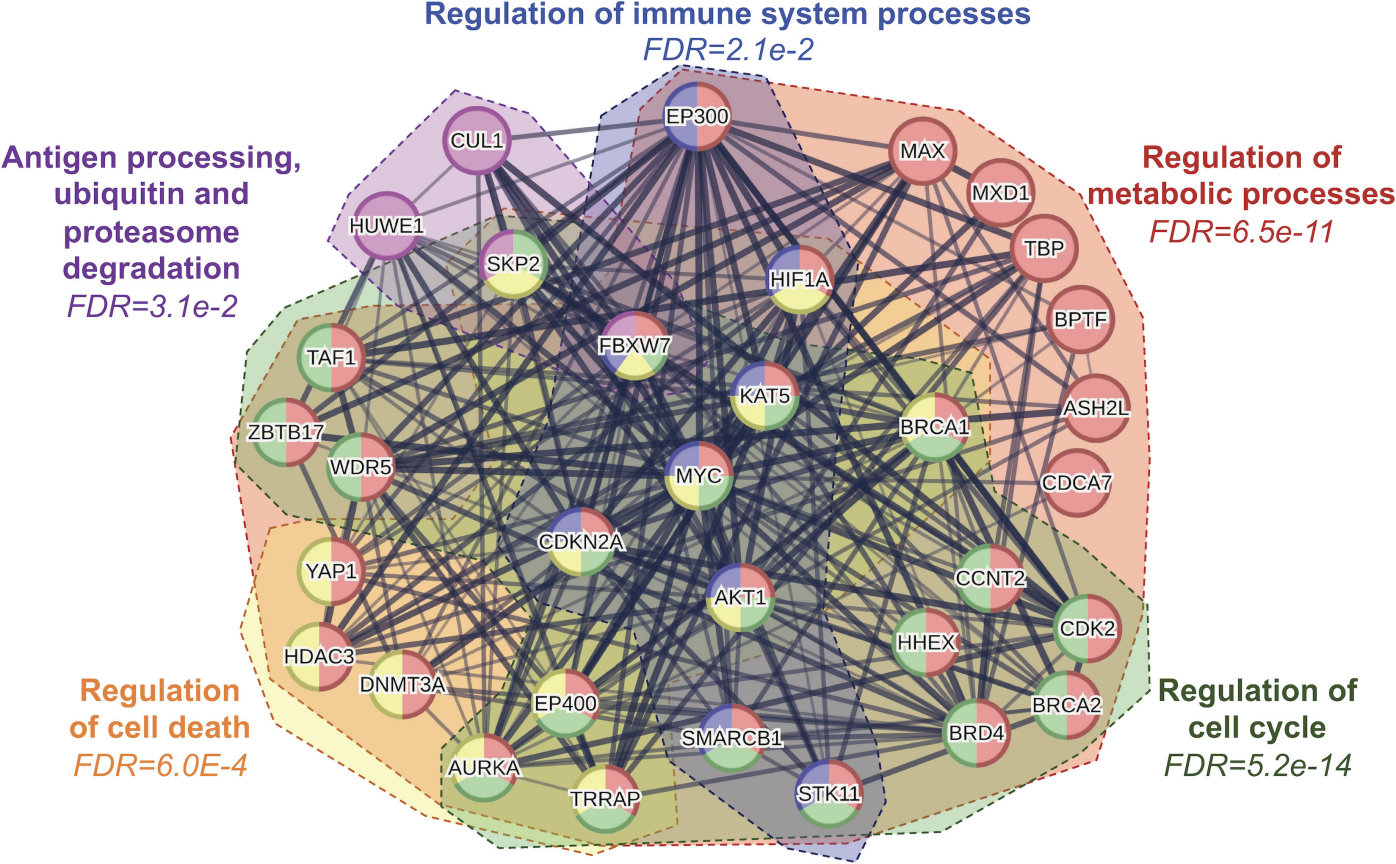
Protein-protein interaction network analysis of MYC and its interacting partners reveal several key regulatory processes including cellular metabolism, immune system, cell cycle and cell death. FDR, false discovery rate.

Of these hallmarks, cancer metabolism and oncoimmunology have garnered interest from several researchers due to the rising opportunities in therapeutic development. In this direction, MYC is a systemic regulator of diverse functions by employing various interactors. The MYC interactome extends further into oncoimmunology and oncometabolism by transcribing or repressing specific gene targets. Key interacting partners, stability partners, cofactors, and gene targets of MYC involved in tumor progression illustrated in **Figure 2**, in which their details are summarized in **Table 1**. Some of these key partners are discussed in the contexts of oncometabolism and oncoimmunology in the next section.

**Table 1:** Key partners and interactors of MYC involved in cancer.

Interactor	Interaction Type	Interaction Site	Role in Cancer	Reference
AKT1	Interaction partner	MB1	Energetic and Metabolic Pathways and Developmental Signaling	(52)
ARF	Interaction partner	Between MB1 and MB2, HLH	Tumor suppressor that inhibits MYC transactivation, proliferation, and transformation.	(53)
ASH2L	Interaction partner	Between MB3b and MB4	Epigenetic regulation	(54)
AURKA	Stability partner	MB1	Promotes tumor invasion, migration, proliferation. Protects MYC from proteasomal degradation	(55)
BPTF	Cofactors	NK^†^	Cancer cell proliferation, cell cycle progression	(56)
BRCA1	Gene Target/Antagonist	–	Tumor suppressor, DNA repair activity	(57, 58)
BRCA2	Gene Target	–	Genomic Instability/DNA repair activity	(59)
BRD4	Cofactors	MB1	Promotes MYC-activated gene transcription.	(60)
CDCA7	Interaction partner	C-Terminus	Tumorigenesis	(61)
CDK2	Stability partner	MB1	Regulates MYC-mediated suppression of senescence.	(62)
CUL1	Gene target	–	Ubiquitin mediated proteolysis and cell cycle progression	(63)
DNMT3a	Transrepression partner	Between MB2 and MB3a	Represses the transcription of cell cycle dependent kinase inhibitors, promoting tumor cell proliferation.	(38)
FOXO	Antagonist	–	Metabolism, adapting to Hypoxia	(64, 65)
FBW7	Stability partner	MB1	Regulates Ubiquitin mediated degradation of MYC. Prevents MYC-activated tumor progression.	(25, 26)
FBX028	Stability partner	MB2, MB4	Promotes Ubiquitination of MYC	(66)
HDAC3	Transrepression partner	MB3a	Binds to MYC to repress *FOXA2* gene transcription, leading to tumorigenesis.	(41)
HHEX	Interaction partner	HLH	Regulates tumor hyperproliferation, metabolism, and transformation.	(67)
HIF1A	Antagonist	–	Metabolism and Proliferation	(68)
HUWE1	Stability partner	Between MB1 and MB2	Promotes Ubiquitination of MYC	(69)
KAT5	Stability partner	Indirect interaction via Ubiquitin-mediated proteolysis	Invasion and Migration	(70)
LKB1	Interaction partner	NK^†^	Energetic and Metabolic Pathways and Developmental Signaling	(52)
MAD	Cofactors	bHLHLZ	Cell Proliferation, Differentiation, Tumorigenesis	(71)
MAX	Heterodimerization partner	bHLHLZ	Proliferation and Tumor Progression	(72, 73)
MIZ1	Interaction partner	bHLHLZ	Tumorigenesis	(44)
p27	Cofactors/Antagonist	MB4	Proliferation and Tumor Progression	(74, 75)
p300	Cofactors	MB4	Proliferation, Invasion and Migration	(23)
p400	Cofactors	MB2	Facilitates Gene Expression of MYC targets	(32)
P65	Antagonist/Transactivation	–	Immune Checkpoint expression, Inhibiting Apoptosis	(76, 77)
p-TEFb	Transactivation partner	MB1	Facilitates Gene Expression of MYC targets.	(28)
SIN3	Stability partner	MB3a	Recruits HDAC1 to exert deacetylase activity. Induces the degradation of MYC.	(78)
SKP2	Stability partner	MB2, HLH	Ubiquitin mediated proteolysis and cell cycle progression	(33)
SNF5	Transactivation	HLH	Facilitates Gene Expression of MYC targets. The protein itself has tumor suppressor roles by suppressing tumorigenesis.	(79, 80)
TAF1	Transactivation partner	Between MB1 and MB2	Essential for forming the transcription initiation complex TFIID, to activate MYC-activated gene transcription.	(27)
TBP	Transactivation partner	Between MB1 and MB2	Essential for forming the transcription initiation complex TFIID, to activate MYC-activated gene transcription.	(27)
TIP48/49	Cofactor	MB2	Essential cofactor for oncogenic transformation induced by MYC activation.	(31)
TIP60	Transactivation partner	MB2	Mediator to recruit Histone Acetyltransferases to MYC to facilitate gene expression of MYC targets.	(30)
TRRAP	Cofactors	MB2	Facilitates Gene expression of MYC targets	(3, 81)
VEGFA	Gene Target	–	Angiogenesis	(82)
WDR5	Interaction partner	MB3b	Tumorigenesis	(83)
YAP1	Interaction partner	NK^†^	Energetic and Metabolic Pathways and Developmental Signaling	(52)

NK^† -^ Interaction Site Not Known; (-) No Physical Interaction.

## 3 MYC roles in oncometabolism and oncoimmunology

The regulatory network of MYC is extensive, spanning across gene targets and cofactors involved in various aspects of cancer development, including cellular metabolism and immunology. Aberrant cell proliferation not only requires altered energy metabolism but also evasion from immunosurveillance. Recent evidence suggests that metabolism is a key element that controls immune evasion (84–86). The following sections summarize the role of MYC in regulating key elements of oncometabolism and oncoimmunology.

### 3.1 MYC and cancer metabolic reprogramming

In the case of regulated cell growth and proliferation, nutrient availability is essential. Hence, there needs to be a system in place to “sense” the level of available nutrients, to regulate the metabolism of available resources and maintain the balance of homeostasis. In mammals, systemically, this regulation occurs with the storage of glucose as glycogen in the liver, and the metabolism of fat by lipolysis, in response to starvation. At a cellular level, the availability of nutrients affects the activation of mTOR, and subsequently MYC expression. In the availability of nutrients, mTOR is activated in cells, which phosphorylates PI3K-AKT and therefore inhibits FOXO, a MYC antagonist (65). The activated mTOR also enhances MYC translation and function in transcribing genes favoring cancer progression (87). However, nutrient shortage inhibits mTOR activation, which thereby yields active FOXO, that limits MYC expression and function (65).

**Figure 3:**
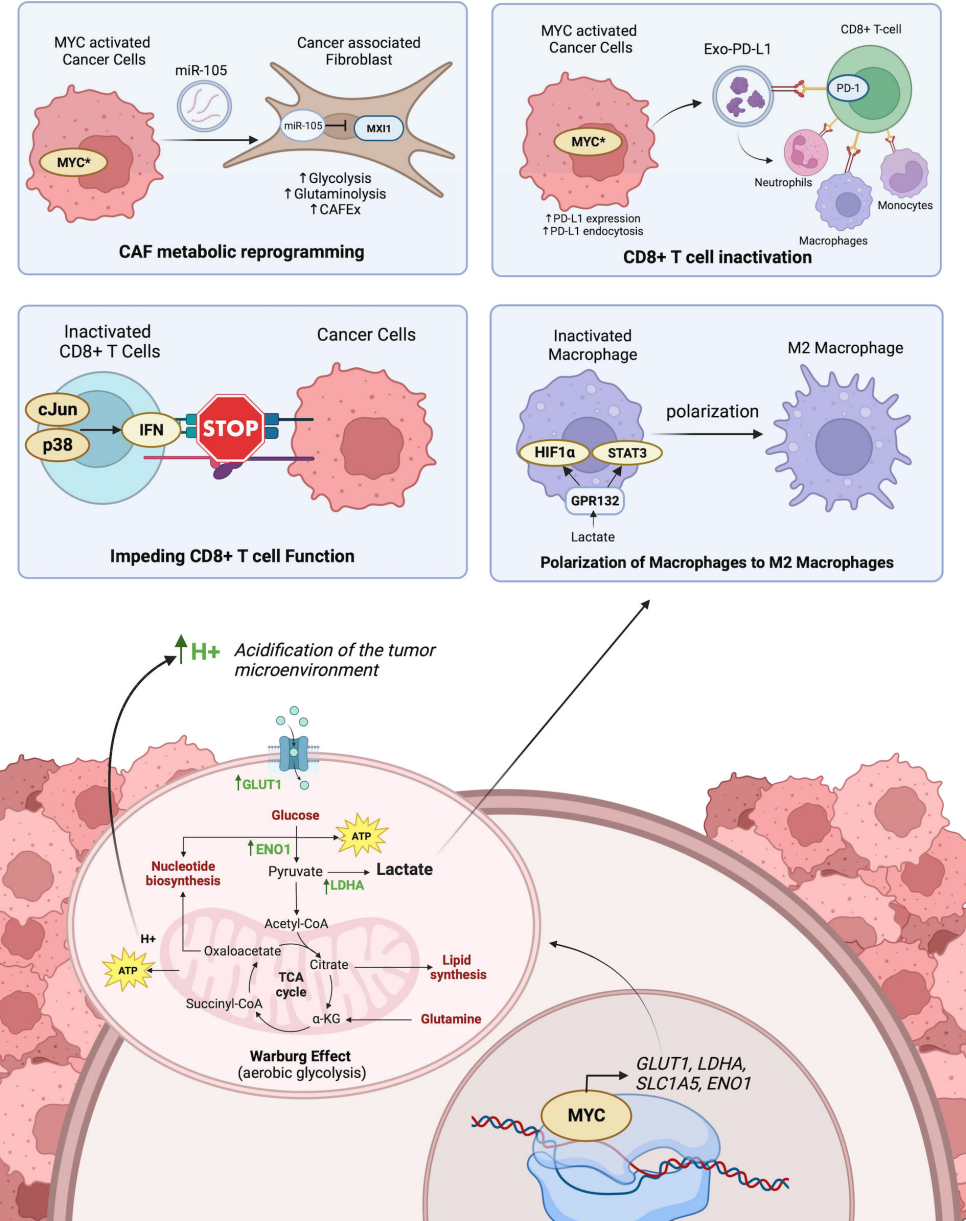
The role of MYC-driven transcriptional activation on cancer and immune cell metabolism and its influence on anti-tumor immunity. Top-left panel shows CAF metabolic reprogramming as a result of activated MYC in tumors exporting miR-105 which is imported into CAFs and inhibits MXI1. Top-right panel shows the inactivation of CD8^+^ T cells by MYC activated export of PD-L1 from tumor cells bound to PD-1 receptors on CD8^+.^ T cells. Middle left panel shows how the acidification of the microenvironment triggers p38 and c-Jun signaling pathways in CD8^+^ T cells which promotes interferon-mediated inactivation of CD8^+^ T cell function. Middle right panel shows lactate released in the tumor microenvironment from tumor cells polarizes the differentiation of M1 macrophages to M2 macrophages. The bottom panel shows how MYC activated transcription of key enzymes promotes Warburg Effect within tumor cells. Created with BioRender.com.

In the process of neoplastic transformation, cancer cells require an increase in glucose uptake to energize their rapid proliferation. Interestingly, this glucose is fermented to produce lactate in the presence of oxygen in a process called the Warburg Effect to yield energy in the form of adenosine triphosphate (ATP). Several investigators revealed that this increased consumption of glucose is due to the oncogenic levels of MYC, as evidenced in Burkitt’s lymphoma (88) and MYC-driven liver carcinoma (89). This occurs by MYC upregulating various elements of the glycolytic cycle, such as the expression of glucose transporter, GLUT1 (90), glycolytic enzymes hexokinase 2 (HK2), phosphofructokinase-M1 (PFKM-1) (91), enolase-1 (ENO1) and lactate dehydrogenase A (LDHA) (92). As a result, the increased glucose uptake and metabolic glycolysis driven by MYC, leads to an accumulation of lactate. While often misconstrued as a waste product, tumors take advantage of the lactate produced by the Warburg Effect to promote various pro-oncogenic functions such as immunomodulation and angiogenesis (93). Consequently, a plausible alternative strategy is to inhibit MYC-driven metabolic reprogramming. For instance, Cargill et al. (94) reported the therapeutic potential of a small molecule inhibitor of a glycolytic enzyme, PFKFB3, in inhibiting the downstream effects of MYC in small cell lung cancer. Moreover, Zuo et al. (95), explored the use of vitamin D activated-long noncoding RNA *MEG3* to suppress glycolysis by promoting c-MYC degradation in colorectal cancer. As mentioned earlier, MYC exerts control over multiple targets within the glycolytic process, these findings support a potential therapeutic approach by targeting specific components that abate MYC-driven glycolysis. The role of MYC in cancer metabolism is depicted in **Figure 3**.

In promoting glucose uptake and metabolism, NAD^+^ ions are produced as metabolites, which are utilized in amino acid synthesis. Cancer cells exhibit a reliance on amino acids, which promote their survival and proliferation, especially under nutritional constraints. Therefore, malignant cells hijack mechanisms to upregulate amino acid production (96).

Just as MYC is a key driver of the metabolic switch in the presence of oxygen (normoxia), HIF1A is a key driver of the metabolic switch in the absence of oxygen (hypoxia) (97). In hypoxic conditions, MYC activity is usually inhibited by HIF1a by impeding the heterodimerization of MYC/MAX complex. HIF1a and MXI1 bind to MAX, thereby yielding unbound MYC destined for degradation (68). This impediment to MYC activity subsequently affects MYC target genes involved in mitochondrial biogenesis, apoptosis, and metabolic reprogramming (98). HIF1A also impedes MYC activity by upregulating the expression of FOXO3a which binds to MYC gene target promoters (99). Notably, however, when MYC is overexpressed, it overcomes the inhibitory effects of HIF1A. Although MYC and HIF1A antagonize each other functions, they share common gene targets in glycolysis, including HK2, PFK1, ENO1, and LDHA. Additionally, when both MYC and HIF1A are overexpressed, they collaborate in promoting angiogenesis and activating the expression of their gene targets (100). Thus, both HIF1A and MYC are key therapeutic targets for cancer progression.

MYC reprograms amino acid metabolism by activating the serine and glutamine synthesis pathways. Under nutrient-deprived conditions, MYC upregulates the expression of five major enzymes in serine biosynthesis, i.e., phosphoglycerate dehydrogenase (PHGDH), phosphoserine aminotransferase 1 (PSAT1), phosphoserine phosphatase (PSPH), serine hydroxymethyltransferases 1 and 2 (SHMT1 and SHMT2). The transcriptional upregulation of these genes facilitates nucleic acid production and cell cycle progression (101, 102). Another amino acid in high demand during tumor development is glutamine. MYC upregulates glutamine synthetase (*GS*) to promote glutamine anabolism (103), and paradoxically, it enhances glutamine catabolism by upregulating SLC1A5 and SLC7A5 amino acid transporters (104). To facilitate the conversion of glutamine to glutamate, MYC upregulates the expression of glutaminase (*GLS)* (105) and represses the expression of miR-23 which interrupts *GLS* translation (106). The availability of amino acids has emerged as a promising therapeutic target. As a result, there has been a significant focus on developing inhibitors that specifically target enzymes involved in amino acid synthesis. For example, pharmacological inhibition of MYC-driven GLS by CB-839 has recently shown encouraging results in suppressing various cancers *in vitro* and *in vivo* (107–109), and currently examined in a phase 1 clinical trial of solid tumors (NCT02071862).

### 3.2 MYC and cancer immune evasion

The immune system is a highly regulated defense mechanism instated to recognize and eliminate pathogens, or dysregulated cells, to maintain a healthy body. As cancer cells propagate uncontrollably, they acquire traits to evade immune recognition. This happens by downregulating self-antigen presentation, promoting an immunosuppressive TME through the release of cytokines, recruiting pro-tumoral immune cells, and increasing the expression of inhibitory immune checkpoint molecules. MYC is reportedly a grand orchestrator of cancer growth and immune evasion, as it regulates most of these traits by modulating its gene targets (110).

In establishing a tumor-proliferative environment beneath the surveillance of anti-tumor immune cells, tumor cells must recruit and modulate regulatory immune cells. In a lung adenoma model *in vivo*, Kortlever et al. (72) revealed that MYC cooperated with KRAS to reprogram stromal cells via epithelial-derived CCL9 and IL-23, resulting in CCL9-mediated macrophage recruitment, PD-L1-dependent discrimination of T and B cells, and IL-23 mediated exclusion of adaptive T and B cells and innate immune NK cells. Deactivating MYC was found to reverse this reprogramming and reinstate normal anti-tumor immune function (111). Noted that MYC is upregulated in tumor-associated macrophages (TAMs), which is involved in suppressing immunosurveillance (112). Moreover, in head and neck squamous cell carcinoma (HNSCC), therapeutic inhibition of MYC promoted intrinsic anti-tumor immune responses through the cGAS-STING signaling pathway, and CD8^+^ T-cell infiltration of HNSCC *in vivo* (113). Together, this evidence shows how MYC creates the TME through the release of cytokines or modulating gene expression to promote pro-tumoral immune cell infiltration and suppress immunosurveillance.

Importantly, MYC also governs the expression of immune checkpoint molecules and self-antigens to switch off immune cell recognition of tumors. Particularly in osteosarcoma, Jiang et al. (114), observed that pharmacological inhibition of MYC resulted in reprogramming the tumor immune microenvironment through the release of T-cell recruiting chemokines and crosstalk of co-stimulatory immune checkpoint molecules CD40 and CD40L. Moreover, a recent study by Dhanasekaran et al. (115), reported that MYC transcriptionally repressed MHC-1 antigen presentation and therefore repressed T-cell immune response in MYC-driven hepatocellular carcinoma. This phenomenon was pharmacologically reversible by the dual-inhibition of immune checkpoint molecules, PD-L1 and CTLA-4. MYC directly regulates the expression of CD47 and PD-L1 through transcriptional activation (116). Other than upregulating the expression of PD-L1 as cell surface receptors, expressed PD-L1 is also packaged into vesicles for export into exo-PD-L1 (117). This exo-PD-L1 promotes immune escape by PD-1/PD-L1 mediated cytotoxic T-cell inactivation through direct interaction or indirectly by exo-PD-L1 uptake in tumor-promoting immune cells (**Figure 3**). Recent investigations have reported evidence of exo-PD-L1 in various cancers including prostate, breast, melanoma, and pancreatic cancer (118). Besides the regulation of immune checkpoint molecule expression, MYC is also involved in post-translational modification of immunosuppressive glycans. Smith et al. (119), recently demonstrated that MYC regulates Siglec ligands through the transcriptional regulation of *ST6GALNAC4* and the induction of a glycan so-called disialylated Galβ1-3GalNAc (disialyl-T antigen). Disialyl-T functions as an inhibitory glyco-immune checkpoint molecule that “switches off” immune response in T-cells by engaging pro-tumoral macrophages. This shows that MYC systemically extends itself in creating an immunosuppressive environment by recruiting pro-tumoral macrophages, repelling anti-tumor immune cells, releasing cytokines, and modulating immune checkpoint molecules, by transcriptionally activating key gene targets.

Cumulatively, MYC systemically extends itself in creating an immunosuppressive environment by recruiting pro-tumoral macrophages, repelling anti-tumor immune cells, releasing cytokines, and modulating immune checkpoint molecules, by transcriptionally activating key gene targets.

### 3.3 MYC at the intersection between oncometabolism and oncoimmunology

Since MYC plays an essential role in regulating key targets involved in both metabolic reprogramming and immune evasion, it is likely that MYC induces one hallmark to influence the activation of another. Studies to date support this notion. **Figure 3** summarizes the role of MYC at the interplay between cancer metabolism and oncoimmunology.

MYC promotes the Warburg Effect by upregulating glucose transporters and key glycolytic enzymes, the yield of H^+^ ions from NADH reduction influences the microenvironment by lowering the pH. This acidic environment facilitates cancer cells to invade the tumor stroma (120). This acidification of the microenvironment also suppresses CD8^+^ T lymphocyte functions, thereby promoting an immunosuppressive microenvironment. More specifically, this is mediated by activation of the p38, JNK/c-Jun signaling pathways, which promotes interferon production (121). Moreover, the lactate produced from tumors polarizes M2-tumor-associated macrophages (122). This is facilitated by the recognition of extracellular lactate levels with GPR132, and the subsequent upregulation of HIF-1α and activation of STAT3 signaling (123, 124). HIF-1α and MYC reciprocally regulate the expression of each. MYC is often seen to interact with HIF-1α in regulating T-cell metabolism by transcriptionally regulating genes involved in glucose and glutamine transport. Moreover, HIF-1α cooperates with MYC to shape the tumor immune microenvironment (100, 125). Similarly, Marchingo et al. (126), unraveled metabolic proteome changes including SLC7A5 and SLC1A5 during T cell activation governed by MYC. These are some of the ways MYC influences oncoimmunology by promoting glucose or amino acid metabolisms.

Conversely, modulating the tumor immune microenvironment also influences cellular energetics. This is particularly evidenced in the metabolic reprogramming of T lymphocytes after antigen activation. Wang et al. (127), reported the antigen activation of T lymphocytes drove the upregulation of genes encoding enzymes and transporters involved in glycolysis and glutaminolysis as governed by MYC. This antigen-activated MYC-driven metabolic reprogramming is responsible for T cell proliferation. Another investigation by Tsai et al. (128) focused on how immunoediting of the TME in early-stage tumorigenesis reprograms cancer metabolism in a way that supports immune evasion. The results suggested that interferon-gamma (IFNγ) released from T cell immunosurveillance stimulated STAT3-dependent MYC upregulation in melanoma cells, which subsequently activated genes involved in glycolysis and oxidative phosphorylation while suppressing IFNγ-induced cellular senescence (128). Besides T cells, cancer-associated fibroblasts (CAFs) are known to play an important role in regulating antitumoral immunity by recruiting the infiltration of effector T cells and modifying immunosuppressive cells (129, 130). CAFs also influence the metabolism of cancer cells through the secretion of various metabolites that fuel cancer proliferation (131). In breast cancer, MYC promotes this interaction through extracellular vesicles (EVs) containing miR-105 transported from cancer cells to CAFs (132). MiR-105 suppresses the expression of endogenous MYC inhibitor MXI1, thereby sustaining MYC activation in CAFs and subsequently facilitating glucose and glutamine metabolisms (132). Increased metabolism in CAFs yields increased lactate levels in the TME which offers an advantage for cancer cells and impedes effector T-cell function (133). While the influence of cancer metabolism on the immunosuppressive TME is well characterized, there are fewer studies exploring the reciprocal communication between cancer and stromal cells through EV molecular cargos.

Besides the dynamic abundance of MYC effecting global transcriptional changes involved in oncometabolism and oncoimmunology, MYC can modulate gene targets that induce metabolic changes influencing cancer immunity and possess dual roles in cancer development. We have compiled the summarized information in **Table 2**, highlighting a few notable gene targets of the exhaustive list of MYC-regulated genes involved in both oncometabolism and oncoimmunology. Further studies focusing on the gene targets and MYC-regulated gene network at the immune-metabolic crossroad shall offer novel alternative strategies to attenuate tumor invasiveness and treatment resistance caused by MYC aberration.

**Table 2:** Key gene targets of MYC in cancer metabolism and oncoimmunology.

Gene Target	Main Hallmark	Role in Oncometabolism	Role in Oncoimmunology	Reference
LDHA	Metabolism	Required in the production of lactate in anerobic glycolysis.	Inhibits immune killing and promotes immunosuppression by increasing lactate production and influencing the microenvironment. Negatively regulates immune infiltration.	(92) (134, 135)
GLUT1	Metabolism	A glucose transporter responsible for the uptake of glucose into cells.	Associated with increases in neutrophil, platelet, monocytes, and lymphocyte count. Negatively correlates with tumor-infiltrating T -cells but positively correlates with neutrophils and dendritic cells	(90, 136, 137)
ENO1	Metabolism	Responsible for converting 3’ biphosphoglycerate to 3’biphosphopyruvate	Promotes anti-tumor immunity by promoting PD-L1 proteolysis.	(90, 138)
SLC1A5	Metabolism	Glutamine Transporter	Overexpression is associated with the presence of immunosuppressive immune cells such as CD68+ macrophage, FOXP3+ regulatory T cells, CD20+ B cells, and PD1+ lymphocytes. SLC1A5 is also required for MYC induction of cytokine-stimulated NK cells.	(84) (139)
SLC38A5	Metabolism	Glutamine Transporter and amino acid coupled Na+/H+ exchanger	Maintains extracellular acidification while maintaining intracellular pH. Acidification of the microenvironment turns off? anti-tumor lymphocyte function.	(140, 141)
IL-23	Immunology	When secreted by tumor-associated macrophages it Interlinks glutamine addiction and immune evasion in kidney cancer.	Cytokine that recruits pro-tumoral macrophages	(111, 142)
CD47	Immunology	Tumor intrinsic CD47 regulates glycolysis in colorectal cancer cells by stabilizing ENO1.	Inhibitory Immune Checkpoint Molecule which turns off immune response in NK and T cells	(143, 144)
PD-L1	Immunology	Regulates glycolysis by improving PFKFB3 expression in renal cell carcinoma cells.	Inhibitory Immune Checkpoint Molecule which turns off immune response in NK and T cells	(143, 145)
VEGF	Immunology	Exogenous VEGF alters metabolism of triple negative breast cancer cells by modulating MAPK-ERK and PI3K-AKT pathways	An immunosuppressive growth factor that impedes the development of T cells and impairs maturation of dendritic cells.	(146–149)
HIF1A	Immunology	Transcribes genes that encode glycolytic enzymes (such as HK2, TPI, ENO1, and PKM) and glutamine metabolism.	Produces IL-9 during TH9 differentiation involved in pro-inflammatory signaling and anti-tumor immunity. HIF1A also partners with mTOR to promote CD8 memory T cell generation. HIF1A also upregulates PD-L1 on tumor cells.	(125, 150–152)
STING	Immunology	STING driven interferon signaling drives metabolic reprogramming of pancreatic cancer cells.	STING induced interferon signaling is crucial in inducing anti-cancer immune response. STING activation enhances antigen presentation and therefore activation of T cells.	(153–155)
TGFB	Immunology	Canonical signaling of TGF-β modulates metabolic reprogramming by upregulating genes involved in glycolysis and oxidative phosphorylation.	TGF-β is a cytokine that promotes cancer progression by impairing T cell proliferation and expansion. TGF-β in cancer associated fibroblasts also promotes immune evasion through ECM signaling.	(156–158)

## 4 Targeting MYC to tackle oncometabolism and oncoimmunology: 2 birds 1 stone?

**Figure 4:**
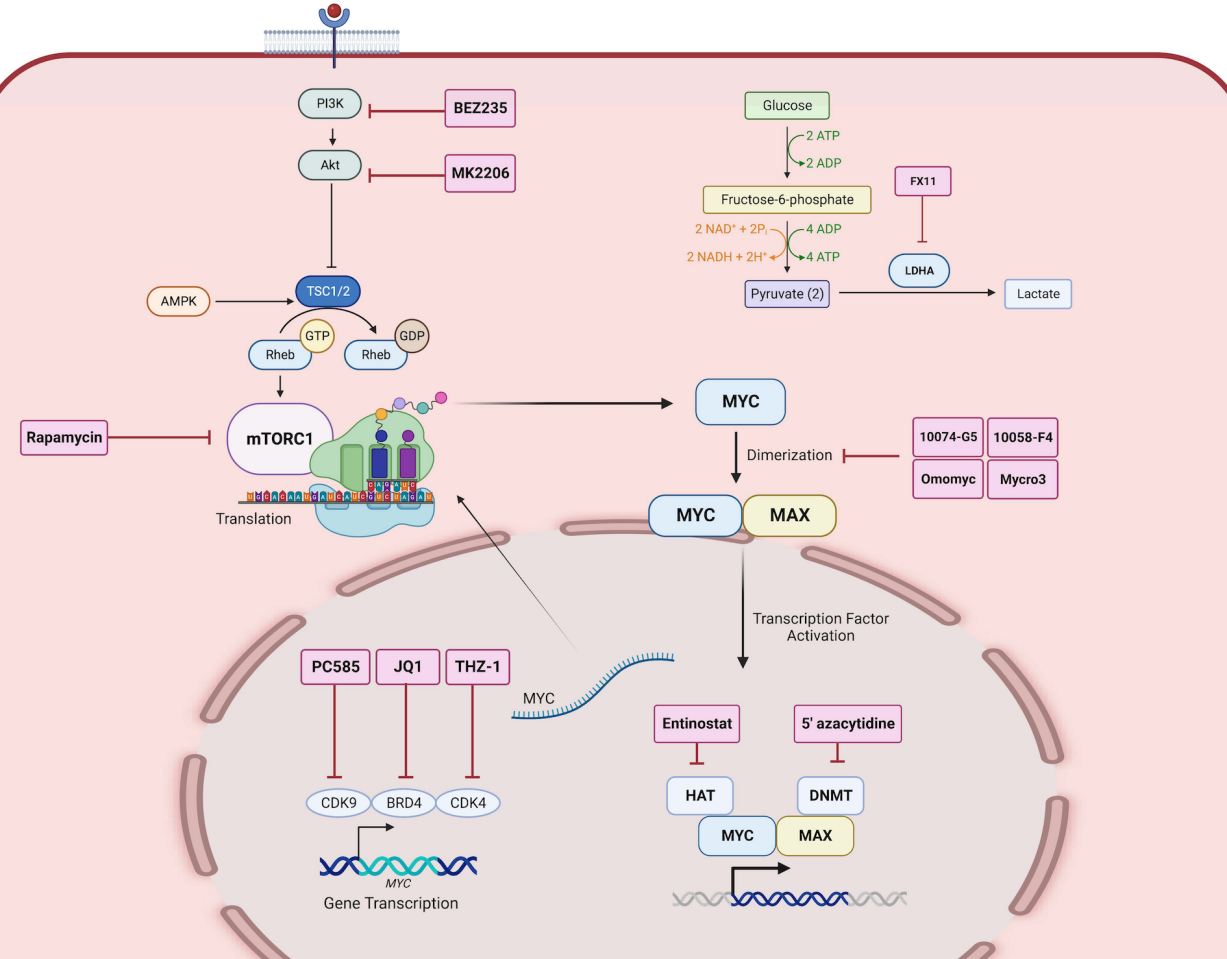
Direct and indirect MYC-targeted therapeutic strategies. Therapeutic inhibitors are depicted as labeled red boxes. PC585 inhibits CDK9, JQ1 inhibits BRD4, and THZ-1 inhibits CDK4, which together are key transcription factors that regulate MYC gene expression. BEZ235 is a PI3K inhibitor, MK2206 is an Akt inhibitor, and Rapamycin is a mTOR inhibitor, which together inhibit the translation of MYC. 10074-G5, 10058-F4, Omomyc, and Mycro3 inhibits the heterodimerization of MYC and MAX. Entinostat inhibits HAT and 5’azacytidine inhibits DNMT which are co-factors that aid in MYC activated transcription of gene targets. FX11 inhibits LDHA, a gene target of MYC, and thus inhibits the downstream function of MYC activation. Created with BioRender.com.

MYC has previously been labeled “undruggable” due to its lack of an enzymatic active site and inaccessibility to its nuclear localization (20, 159). Various approaches have been employed to address the undruggable MYC through its actionable interacting partners and gene targets as illustrated in **Figure 4**.

Investigators exploited the heterodimerization between MYC and MAX to inactivate MYC-activated transcription. One study showed that pharmacological inhibition of MYC by 10058-F4 resulted in changes in lipid and amino acid metabolism in neuroblastoma cell lines (160). Additionally, another MYC-MAX perturbagen, Mycro3 resulted in enhanced CD8^+^T cell function in surveilling cancer cells and inducing anti-tumor immune response (161). Another approach is the inhibition of MYC transcription by bromodomain-containing 4 (BRD4), using inhibitors such as JQ1 and OTX-015 (162). In medulloblastoma, the transcriptional inhibition of MYC by OTX-015 alters cancer glycolysis and amino acid metabolism (163). Moreover, the use of JQ1 in neuroblastoma, melanoma cells promoted tumor immunogenicity and potentiated immune checkpoint blockade therapy (164). However, over the decades, MYC-targeted strategies against cancers have yet to see success in clinical trials due to the half-life of MYC and the rapid metabolism of the small-molecule inhibitors (20, 165). One significant challenge has been translating *in vitro* findings *in vivo* (166), until recently.

In the advent of overcoming the limitations of current MYC inhibitor designs, Omomyc, a 90 amino acid mutant MYC peptide that disrupts the MYC-MAX dimerization, rose to clinical development (167). Omomyc has exuded various pro-apoptotic effects in various cancers, and the potential of immune reprogramming of tumors (168). However, the effect of Omomyc treatment on the metabolic reprogramming of cancers is yet to be determined. Because of its potent reduction of tumor burden, Omomyc stands as the first direct MYC inhibitor to ascend in dose-escalated phase 1 and phase 2 clinical trials of patients with non-small cell lung, colorectal, and breast cancer (NCT04808362). More recently, another phase 1 clinical trial (NCT06059001) has been initiated in metastatic pancreatic cancer. This success should encourage further improvements in this design to effectively target MYC and systemically shut down MYC-driven oncogenic pathways.

The growing body of evidence of the vastness of the “onco-MYC network” and its grave implications on cancer progression point to MYC being an ideal therapeutic target. Considering the overlap in function of the gene targets of MYC between oncometabolism and oncoimmunology, we believe that targeting MYC directly or indirectly may systemically impact both hallmarks. Several investigators have untangled the MYC network to identify indirect putative targets to combat MYC-driven effects. For example, the inhibition of LDHA, a direct gene target of MYC, by FX11, not only suppresses MYC but also inhibits MYC-induced metabolic changes (169). Moreover, inhibition of MYC-regulated glutaminase (GLS) by CB-839 also has a similar effect in reversing MYC-driven metabolic changes such as nucleotide metabolism in ovarian and glioblastoma (109, 170). Moreover, this has shown promise for clinical development in various cancers including colorectal and leukemic cancers (NCT02861300; NCT02071927).

The approach of tackling MYC gene targets has also been successful in modulating the immune evasive nature of tumors. For instance, dual inhibition of MYC targets PD-L1 and CTLA-4 reverses MYC-driven immunosuppression through pro-inflammatory macrophages in hepatocellular carcinoma (115). Moreover, MYC partners with epigenetic modulators such as histone acetylases (HAT) and DNA methylases (DNMT), in the transcriptional activation of immunosuppressive gene targets of MYC (171). Targeting, MYC-epigenetic modulators may reverse this phenomenon and exude anticancer effects. In this direction, Topper et al. (172), tested this hypothesis by combining epigenetic modulators including 5’-azacytidine and entinostat to assess its effect on tumor burden. As a result, this combination increased the number CD8^+^T and natural killer cells in the TME, promoted immunosurveillance of tumors, and reduced MYC-driven interferon signaling. These indirect pharmacological inhibitions are effective in modulating the downstream effects of MYC-driven tumors (172). These indirect pharmacological inhibitions are effective in modulating the downstream effects of MYC aberration as aforementioned, the therapeutic potentials of targeting gene targets at the crossroad between oncometabolism and oncoimmunology (**Table 2**) warrants further investigations.

## 5 Challenges and perspectives

Despite the success of Omomyc in preclinical models, the development of MYC-targeted therapy has miles to go until we reach the growing demand of patients who require effective treatment. The main challenge posed against all small molecule inhibitors against MYC is the rapid metabolism of the drug, and the quick half-life of MYC regeneration. One reported limitation of Omomyc is the fast distribution and catabolism, thereby limiting its use in preclinical and *in vivo* models (173). Other challenges include the multiple disordered conformations of the putative binding regions of MYC (174). Thus, this warrants further development in the design of MYC inhibition. Recent investigations approach this issue by using *in silico* tools to facilitate drug design. Using *in silico* tools offers a wealth of information to guide the development of a MYC-targeted therapeutic strategy. This ranges from identifying potential binding sites on MYC and predicting different drug binding conformations using molecular docking to identifying close targets or partners upstream or downstream of MYC. For instance, Yu et al. employed conformational simulation of intrinsically disordered MYC to identify binding sites and “multi-conformational” molecular docking. This guided the identification of seven compounds that bind to MYC *in vitro* and inhibited cell proliferation in *c-MYC* overexpressing cell lines (175). Moreover, in 2018, a novel inhibitor, 7594-0035 was reported to specifically target MYC indicated for the treatment of refractory multiple myeloma. The novel inhibitor was identified using the drug database ChemDiv and molecularly docked to the crystallized structure of the MYC-MAX heterodimer complexed with DNA (PDB ID: 1NKP) (176). This evidence shows promise in unmasking the elusive binding pockets of MYC by simulating the interaction between the MYC-MAX heterodimer and small molecule structures, to develop better direct inhibitors of the MYC oncoprotein.

The advent of machine learning and artificial intelligence opens opportunities for investigators to design novel peptides, predict novel binding sites on MYC, and explore indirect key partners or regulators of MYC that may be therapeutically targeted alternatively. One successful example of this approach is the discovery of novel inhibitors by Xing et al. (177). that target BRD4 which regulates the transcription of the *MYC* gene (**Figure 4**). In their investigation, a structure-based virtual screening approach with machine-learning algorithms was performed to learn the structure of the BRD4 protein and predict the likelihood of the compound inhibiting BRD4 based on its binding pattern. This led to the discovery of 15 new BRD4 inhibitors which were experimentally validated (177). This approach could be extended by integrating machine learning and molecular docking to identify binding pockets within MYC at which predicted inhibitor structures may bind. Another approach utilizes novel *in silico* tools to predict miRNAs capable of regulating MYC and its partners; nonetheless, only a few miRNA regulators of MYC expression, such as miR-19, have been validated (178). This presents an avenue of research yet to be claimed to expand the available therapeutic options for inhibiting MYC.

A promising approach in employing *in silico* tools to discover pharmacological inhibition of MYC is using pharmacogenomic connectivity analysis of cancer transcriptomes and drug sensitivity data. To this effect, the iLINCS consortium facilitates “pharmaco-multi-Omics” analysis by integrating data from transcriptomic, proteomic, phospho-proteomic, and genomic sources to drug sensitivity data from chemical perturbation or gene knockdown signatures (179). This approach may not only supplement our understanding of the potential interactors of MYC, but also of the potential mechanism of action of these small molecules against MYC. An example of this approach being successful is seen in an excellent investigation led by Howard et al. (180). In the interest of repositioning pharmacological inhibitors toward the inhibition of eIF4A1 against triple-negative breast cancer, Howard et al. (180) surveyed and screened the Prestwick Chemical Library for potential therapeutics against eIF4A1, where iLINCS pharmacogenomics was implemented to elucidate the mechanism of action of these candidate molecules. They identified that in the inhibition of eIF4A1, c-MYC is also suppressed, thus warranting further exploration of the interaction between eIF4A1 and c-MYC (180). While this investigation showed how c-MYC itself is an indirect target of some small molecules, future investigations may build on this information and identify other small molecules that impede c-MYC activity.

## 6 Conclusion

MYC activation is characteristic of various aggressive tumor types. This aggression is typically mediated by the crosstalk of cancer metabolism and cancer immunity. MYC is central to both hallmarks by partnering with various cofactors or transcription factors and by its gene targets. This thus presents MYC as a promising therapeutic target for cancer therapy. This review explores how MYC bridges these hallmarks by inducing metabolic reprogramming that influences an immunosuppressive microenvironment, and conversely, promoting immune evasive markers to influence immune cell and cancer cell metabolism. Moreover, the gene targets of MYC are often seen to be involved in both hallmarks and would therefore present as ideal alternative targets to combat MYC-driven effects. Direct inhibition of MYC has been challenging due to the short half-life of MYC oncoprotein and the high metabolism of the small molecule inhibitors, which has impeded the development of MYC inhibitors in clinical trials. However, Omomyc overcame these limitations, exuded potent anti-cancer effects, and has ascended toward clinical development for multiple cancers. This review surmises that MYC inhibition would be beneficial in systemically combating metabolic reprogramming and immune evasion in various cancers. Thus, we encourage more pharmacological strategies should be centered around MYC inhibition. Moreover, future investigation attention should be drawn toward elucidating the molecular mechanism behind MYC inhibition in both oncometabolism and oncoimmunology.

## Author contributions

SV: Data curation, Methodology, Visualization, Writing – original draft. BB: Data curation, Methodology, Writing – review & editing. CT: Resources, Supervision, Writing – review & editing. JM: Resources, Supervision, Writing – review & editing. RT: Funding acquisition, Resources, Supervision, Writing – review & editing. SC: Conceptualization, Methodology, Supervision, Visualization, Writing – review & editing.
